# Translational relevance of animal models available on brain arteriovenous malformations, a systematic review

**DOI:** 10.1177/0271678X251409038

**Published:** 2026-02-24

**Authors:** Sara Keränen, René Aquarius, Juhana Frösen, Carlijn R Hooijmans, Hieronymus D Boogaarts

**Affiliations:** 1 Hemorrhagic Brain Pathology Research Group, Tampere University, Tampere, Finland; 2 Department of Neurosurgery, Tampere University Hospital, Tampere, Finland; 3 Department of Neurosurgery, Radboud university medical center, Nijmegen, The Netherlands; 4 Department of Anesthesiology, Pain and Palliative Medicine, Radboud university medical center, Nijmegen, The Netherlands

**Keywords:** Animal model, brain arteriovenous malformation, intracranial hemorrhage, KRAS mutation, systematic review

## Abstract

We conducted a systematic review to identify and evaluate animal models of true brain arteriovenous malformations (bAVMs), focusing on how they replicate human disease. Outcomes assessed included bAVM growth, rupture, seizures, and survival. The review adhered to PRISMA guidelines and SYRCLE’s protocol for animal studies. A search on PubMed and Embase was conducted (April 19, 2023; updated October 24, 2024). Exclusion criteria were; (1) Not an original, peer-reviewed full length research article, (2) not an in vivo study, (3) no bAVM induction, (4) no appropriate control group, (5) no histological or anatomical assessment during the bAVM follow-up, (6) outcome of the bAVM not reported. Meta analyses were planned for all key parameters. Risk of bias and image duplication assessments were conducted. Forty-one studies could be included in this SR. Models primarily involved mice and targeted mutations in MAPK, TGFbeta and Notch related signaling. Study designs varied significantly, limiting meta-analysis and direct comparison. We often noted high risk of bias in studies’ reporting. Many studies had high risk of bias and focused on HHT-related mutations, which represent only a minority of clinical bAVM cases. KRAS-based models may offer better clinical relevance, but overall, current bAVM models show substantial variability.

## Introduction

Brain arteriovenous malformations (bAVM) are high flow vascular anomalies with a direct blood flow from arteries to veins trough a pathological nidus.^1,2^ BAVMs are the primary cause for a non-traumatic intracranial hemorrhage in young adults and cause significant morbidity and mortality.^2,3^ Incidence of bAVMs is reported to be 1.12–1.42 per 100,000 person-years. BAVMs may remain asymptomatic throughout a person’s lifetime, yet they can also rupture unexpectedly after decades of silence.^4–12^ If symptoms do arise due to a bAVM they usually manifest as epileptic seizures and neurological deficits.^2,13–15^ It is not known why some bAVMs rupture and some remain stable. All of the available therapies for bAVMs (surgery, stereotactic radiosurgery and endovascular embolization) have a significant risk of complications and safer medical therapy options are therefore needed.^16,17^

**Figure 1.**
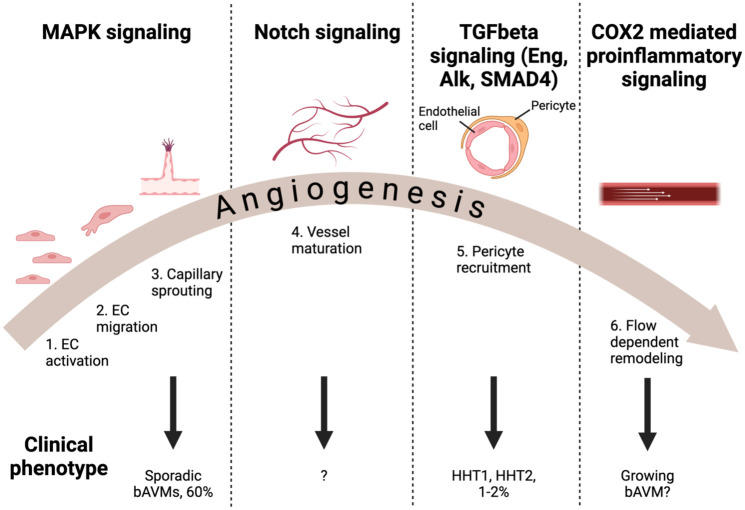
Relevant molecular signaling pathways in bAVMs, and their effects on angiogenesis and the clinical course of the bAVM. EC: endothelial cell.

Various genetic mutations are playing a role in the formation of bAVMs, which have been summarized in Table 1 and Figure 1. Due to different etiologies and affected molecular pathways, not all bAVMs represent the same disease. Most bAVMs are explained by overactivation in MAPK signaling pathway and impaired endothelial cell activation, migration and sprouting, while others are caused by mutations leading to defective TGFbeta or Notch signaling (Figure 1) that control endothelial cell homeostasis and regulation as well as pericyte recruitment and vessel maturation. Therefore, when exploring medical treatment options for patients, it is unlikely that all bAVMs will respond uniformly. It is also possible that the clinical course of the disease might vary depending on the mutation profile explaining the lesion.

**Table 1.** Mutations used in bAVM animal models and seen in bAVM patients.

Mutation	Role	Mutation causing bAVMs in an animal model	Mutation seen in AVM patients	Mutation seen in clinical syndromes that may include bAVMs
KRAS	Overactivation of MAPK signaling pathway	Yes	Yes^18^	No
MAP2K1	Overactivation of MAPK signaling pathway	Yes	Yes^19^	No
BRAF	Overactivation of MAPK signaling pathway	Yes	Yes^20^	No
GPRASP1	Overactivation of MAPK signaling pathway	Yes	Yes^21^	No
HRAS	Overactivation of MAPK signaling pathway	Yes	Yes, extracranial^22^	No
Eng	Loss of function in TGFbeta signaling	Yes	Yes^23^	Yes: HHT1^23^
Alk1	Loss of function in TGFbeta signaling	Yes	Yes^23^	Yes: HHT2^23^
SMAD4	Loss of function in TGFbeta signaling	Yes	Yes^23^	Yes: HHT-juvenile polyposis^24^
MGP	Alk1 overactivation	Yes	No	No
BMP9	Alk1 hypoactivation	Yes	Yes, extracranial^24^	Yes: HHT-like syndrome^24^
Notch1	Overactivation of Notch signaling	Yes	Yes^25^	Yes: Adams-Oliver syndrome^25^
Notch4	Overactivation of Notch signaling	Yes	Yes^25^	Yes: Adams-Oliver syndrome^25^
Shh	Overactivation of Notch signaling	Yes	Yes^26^	No
RBPJ	Overactivation of Notch signaling	Yes	No	Yes: Adams-Oliver syndrome^27^

Alk1: activin receptor-like kinase 1; AVM: arteriovenous malformation; bAVM: arteriovenous malformation in the brain; BMP9: bone morphogenetic protein 9; BRAF: B-Raf proto-oncogene serine/threonine kinase; Eng: endoglin; GPRASP1: G-protein coupled receptor-associated sorting protein 1; HHT: hereditary hemorrhagic teleangiectasia; HRAS: Harvey rat sarcoma viral oncogene homolog; KRAS: Kirsten rat sarcoma viral oncogene homolog; MAPK: mitogen-activated protein kinase; MAP2K1: mitogen-activated protein kinase kinase 1; MGP: matrix Gla protein; Notch1: neurogenic locus Notch homolog protein 1; Notch4: neurogenic locus Notch homolog protein 4; RBPJ: recombination signal binding protein for immunoglobulin kappa J region; Shh: sonic hedgehog signaling molecule; SMAD4: Mothers against decapentaplegic homolog 4; TGFbeta: transforming growth factor beta.

The clinical syndromes potentially involving bAVMs are presented.

We performed a systematic review of the existing literature in order to identify all available animal models on true brain arteriovenous malformations, and to analyze their characteristics in relation to human situation. The most important outcome parameters we will be evaluating are bAVM growth, rupture rate, epileptic seizures and survival.

## Methods

### Reporting and protocol

The aim of this systematic review was to identify and evaluate the animal models that have been developed to study bAVMs. This review is reported according to the PRISMA guidelines.^28^ The review methodology was registered a priori in PROSPERO (protocol ID: CRD42020181676). Three amendments to the review protocol were made during the study: (1) exclusion criterium 1 was expanded and now also explicitly stated that we would exclude articles that were not peer-reviewed; (2) all included articles were screened for the presence of image duplication using a specialized tool and excluded for data extraction and meta-analysis if inappropriate image duplication errors were present; (3) identifying characteristics for individual bAVM models were broadened in order to perform a meta-analysis.

### Search strategy

A comprehensive search was conducted on April 19th, 2023 in PubMed and Embase. The search was updated in Pubmed on October 24th, 2024. The full comprehensive search strategy is presented in Supplemental Material. No restrictions based on publication language or date were applied.

### Study selection

Duplicates were removed and search results were screened using the online screening tool Rayyan (Rayyan Systems, Inc.). In the first phase, papers were screened based on title and abstract. Eligible papers were then screened for inclusion based on the full text. Two reviewers (S.K. and R.A. or H.B.) independently screened the references in both phases. Discrepancies during the screening process were resolved by discussion or by consulting a third co-author (C.H.) if discussion did not resolve the issue. In both phases a paper was excluded if one of the following exclusion criteria was applicable:

1. Not an original, peer-reviewed, full length research article,
2. Not an in vivo study,
3. No bAVM induction (no arteriovenous shunt present in the brain of the animal),
4. No appropriate control group (e.g. healthy, sham-operation),
5. No histological or anatomical assessment during the bAVM follow-up (damage visible on imaging (hemorrhage), damage visible on gross examination, histological analysis)
6. Outcome of the bAVM not reported (survival of the animals, bAVM rupture status, growth status of the bAVM, epilepsy/seizures)

Regarding exclusion criterium 2, we excluded surgically created shunts (i.e. internal carotid artery to external jugular vein) and anatomical features of certain animals (i.e. rete mirabile in porcine animal models) from our systematic review as these models did not realisitically mimic bAVM pathology. If a full text document was not available online, the corresponding author was contacted by email. If this elicited no response within 2 weeks, a reminder was sent. The article was excluded from our review if the authors did not respond to the reminder email within another 2 weeks.

### Image duplication assessment

All articles included after full text screening, were assessed by one author (R.A.) using Imagetwin (Imagetwin AI GmbH, Austria): an AI-based software tool for detecting duplicated figure elements within an article and between the selected article and a Imagetwin-curated database of 75 million scientific figures.^29^ Potential issues flagged by Imagetwin were confirmed by one author (R.A.) and were subsequently discussed between all authors and when all authors agreed that there was a possible serious issue present, it would be described in a PubPeer.com post and the integrity offices of the publisher(s) would be notified. If an article would be retracted or was still under investigation by the publisher, we would not extract any data and no risk of bias assessment would be done.

### Data extraction

Data was extracted by two reviewers (S.K. and R.A or H.B.) and discrepancies were resolved through discussion or by consulting a third co-author (C.H.).

Several data items were extracted from each publication selected to the final analysis such as bibliographic details (first author, year of publication, publication language) and study design details (group sizes, animal species, sex, strain, weight/age and diet and housing conditions). Different animal models were categorized based on the method bAVMs were induced. Regarding the induction method, we extracted time-to-onset, bAVM location, bAVM size, follow-up time and penetrance as well as possible additional triggering factors (e.g. VEGF). When the model was induced with intracranial injections, this was concerned as a method to induce the model, not as an external stimulation. In terms of outcomes, we extracted whether studies included a histological or anatomical assessment (Y/N) (e.g. imaging, immunohistochemical stainings performed, presence of hemorrhage or hemosiderin), bAVM growth status (Y/N) and the presence of epileptic seizures (Y/N). We extracted raw event data from 2 × 2 contingency tables to derive survival and rupture proportions in both the intervention and comparator arms if available

### Risk of bias assessment

All included studies were assessed using SYRCLE’s risk of bias tool^30^ with the addition of the following five reporting quality questions,^31,32^:

1. Was any randomization reported at any level of the experiment? (Y/N)
2. Was any blinding reported at any level of the experiment? (Y/N)
3. Was a power or sample-size calculation reported? (Y/N)
4. Was a conflict of interest statement reported? (Y/N)
5. Was a prespecified / preregistered protocol reported? (Y/N)

**Figure 2.**
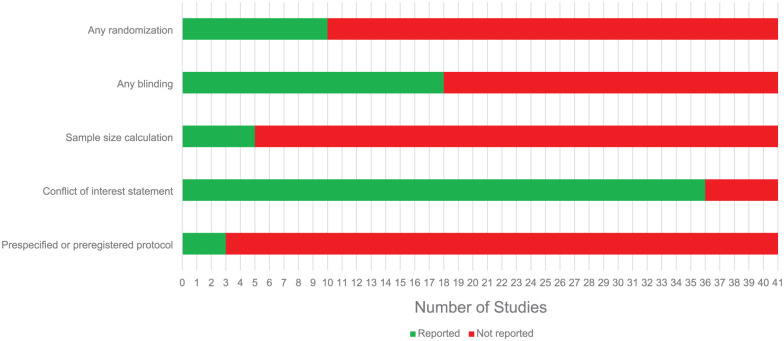
Quality of reporting.

**Figure 3.**
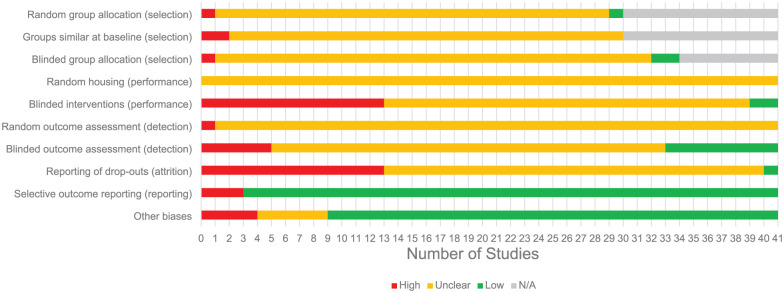
Risk of bias.

Risk of bias was assessed by two independent reviewers (S.K. and R.A. or H.B.). Both reviewers resolved discrepancies through discussion. If no consensus could be achieved, the opinion of a third author (C.H.) would be leading (Figures 2 and 3). Score indications were ‘low’ for low risk of bias, ‘high’ for high risk of bias and ‘unclear’ for unknown risk of bias.

### Data synthesis

We performed meta-analyses comparing all available bAVM animal models as categorized according to the causative genetic mutation (Alk1, Eng, KRAS, Notch). The extracted data was analyzed using the software comprehensive meta-analysis (CMA). For all studies investigating survival and rupture for which also a control group was present we first calculated the OR. In case of zero events or 100% events, we added 0.5 to each cell of the contingency table. Subsequently we conducted meta-analyses. Despite anticipated heterogeneity, the individual effect sizes were pooled to obtain an overall hedges OR and a 95% confidence interval (CI). We used the random effects model, which takes into account the precision of individual studies and the variation between studies and weights each study accordingly. *I*^2^ was used to determine the level of between study heterogeneity. Subgroup analyses were planned for individual bAVM models. We also conducted one armed meta-analyses for all studies investigating survival and rupture status, that did not present data of an control group. Event rates were calculated and pooled using the random effects model.

## Results

### General aspects

The outcome data is presented at Tables 2 and 3. No spontaneous bAVMs were seen in control animals.

**Table 2.** Animal model characteristics.

Model	Mutation	Stimulation or genetic variation	Reference	Species	Strain	Sex	Induction age/weight	Number of animals
MAPK signaling								
	KRAS G12D	N/A	Fish et al.^33^	Mouse	FVB	Both	2–4 months	19
	KRAS G12D	ibEC	Fish et al.^33^	Mouse	FVB	Both	2–4 months	28
	KRAS G12D	N/A	Nguyen et al.^48^	Mouse	Mixed	Both	P1–P3	43
	KRAS G12V	N/A	Park E et al.^49^	Mouse	C57BL/6	Both	5 weeks	11
	HRAS V12	N/A	Li et al.^34^	Mouse	N/R	Both	6–8 weeks	N/R
	MAP2K1	N/A	Smits et al.^50^	Mouse	C57BL/6	N/R	P1	14
	MAP2K1	N/A	Sudduth et al.^51^	Mouse	C57BL/6	N/R	P1	N/R
	GPRASP1	N/A	Li et al.^21^	Mouse	C57BL/6J	N/R	P3–P5	45
	BRAF+/−	N/A	Tu et al.^47^	Mouse	C57BL/6J	N/R	6 weeks	10
	BRAF−/−	N/A	Tu et al.^47^	Mouse	C57BL/6J	N/R	6 weeks	10
HHT1								
	Eng+/−	N/A	Satomi et al.^63^	Mouse	129/Ola, C57BL/6	Both	Developmental	10
	Eng+/−	VEGF	Xu et al.^35^	Mouse	C57BL/6	N/R	8–10 weeks	9
	Eng−/−	Eng2fl/2fl;SM22a-Cre	Choi et al.^36^	Mouse	N/R	Both	Developmental	20
	Eng−/−	VEGF	Choi et al.^36^	Mouse	N/R	Both	8–10 weeks	6
	Eng−/−	VEGF	Choi et al.^37^	Mouse	C57BL/6	N/R	8–10 weeks	6
	Eng−/−	N/A	Han et al.^52^	Mouse	C57BL6, 129Sv	N/R	P1–P3	24
	Eng−/−	N/A	Han et al.^52^	Mouse	C57BL6, 129Sv	N/R	P8–P10	7
	Eng−/−	N/A	Han et al.^52^	Mouse	C57BL6, 129Sv	N/R	P15–P17	11
	Eng−/−	VEGF stimulation, PdgfbicreER	Shabani et al.,^39^ Cells	Mouse	N/R	Both	8–10 weeks	7
	Eng−/−	VEGF stimulation, PdgfbicreER	Shabani et al.,^38^ Biomedicines	Mouse	C57BL/6	Both	8–10 weeks	N/R
HHT2								
	Alk1+/−	VEGF	Chen et al.^40^	Mouse	C57BL/6	N/R	8 weeks	6
	Alk1+/−	VEGF	Hao et al.^41^	Mouse	N/R	Male	8–10 weeks	N/R
	Alk1iECKO	VEGF	Chen et al.^17^	Mouse	C57BL/6	Both	8 weeks	12
	Bmp10-iKO, Bmp9/10 dKO	N/A	Choi et al.^53^	Mouse	C57BL/6J, 129Sv	Both	P2–P3	20
	Alk1−/−	VEGF	Choi et al.^37^	Mouse	C57BL/6	N/R	8–10 weeks	6
	Alk1−/−	N/A	Han et al.^68^	Mouse	C57BL/6J, 129Sv	Both	N/R	55
	Alk1−/−	VEGF	Walker et al.^43^	Mouse	C57BL/6	N/R	8–10 weeks	N/R
	Alk1−/−	VEGF	Walker et al.^44^	Mouse	C57BL/6	N/R	8–10 weeks	18
	Alk1−/−	Flox-SM22Cre-Del	Milton et al.^64^	Mouse	29Sv, C57BL/6, B6SJLF2	Both	Developmental	44
	Alk1−/−	N/A	Park H et al.^49^	Mouse	Mixed	N/R	P4	8
	Alk1−/−	Bone marrow from Alk1 deleted mice into WT mice + AAV injection 4 weeks later + tamoxifen 2 weeks later	Shaligram et al.^45^	Mouse	C57BL/6J	N/R	12 weeks	N/R
	Alk1−/−	VEGF stimulation, PdgfbicreER	Shabani et al.,^39^ Cells	Mouse	N/R	Both	8–10 weeks	7
	Alk1−/−	VEGF stimulation	Shabani et al.,^39^ Cells	Mouse	N/R	Both	8–10 weeks	5
	Alk1KO	N/A	Scherschinski et al.^55^	Mouse	129Sv/C57BL6	Both	P1	59
	CRISPR/Cas9 system to delete Alk1	VEGF	Zhu et al.^46^	Mouse	C57BL/6	Both	8 weeks	12
	MGP−/−	N/A	Marin-Ramos et al.^65^	Mouse	C57BL/6	N/R	Developmental	41
	MGP−/−	N/A	Yao Y et al.^66^	Mouse	C57BL/6	N/R	Developmental	N/R
	MGP−/−	N/A	Yao J et al.^67^	Mouse	C57BL/6	N/R	Developmental	N/R
	Smad4−/−	N/A	Kim Y et al.^56^	Mouse	129Sv, C57BL/6	N/R	P1	26
NOTCH								
	Tie2-tTA;TRE-Notch1	N/A	Murphy et al.^57^	Mouse	N/R	N/R	P1	5
	Tie2-tTA;TRE-Notch4	N/A	Kim TN et al.^58^	Mouse	FVB/N	N/R	P1	3
	Tie2-tTA;TRE-Notch4	N/A	Murphy et al.^57^	Mouse	N/R	N/R	P1	34
	Tie2-tTA;TRE-Notch4	N/A	Murphy et al.^59^	Mouse	N/R	N/R	P1	107
	Tie2-tTA;TRE-Notch4	N/A	Murphy et al.^25^	Mouse	N/R	N/R	P1	3
	Tie2-tTa;TRE-Notch4	N/A	Huang et al.^60^	Mouse	FVB/N	Both	P1	N/R
	Notch4	N/A	Nielsen et al.^69^	Mouse	Mixed	N/R	N/R	10
	Shh	N/A	Giarretta et al.^26^	Rat	Wistar	N/R	250g	5
	Rbpj−/−	N/A	Nielsen et al.^61^	Mouse	N/R	N/R	P1	43
	Rbpj−/−	N/A	Adhicary et al.^27^	Mouse	C57BL/6, FVB/N	Both	P1–P2	N/R
	Rbpj−/−	N/A	Chapman et al.^62^	Mouse	N/R	Both	P1–P2	12

N/A: not applicable; N/R: not reported; ibEC: induced in brain endothelial cells; fl: floxed; KO: knocked-out; iECKO: induced knock-out in endothelial cells; WT: wild type; AAV: adeno-associated virus.

**Table 3.** Animal model outcome measures.

Model	Mutation	Stimulation or genetic variation	Reference	Induction age/weight	Penetrance (%)	Location	Rupture	BAVM growth	Seizures	Survival (%)
MAPK signaling	KRAS G12D	N/A	Fish et al.^33^	2–4 months	66.7	Cortex	0.0%	N/R	N/R	100.0
	KRAS G12D	ibEC	Fish et al.^33^	2–4 months	54.5	Cortex	3.6%	N/R	N/R	100.0
	KRAS G12D	N/A	Nguyen et al.^48^	P1–P3	10.0	N/R	3.8%	N/R	N/R	10.0
	KRAS G12V	N/A	Park E et al.^49^	5 weeks	100.0	Forebrain, basal ganglia	N/R	N/R	N/R	N/R
	HRAS V12	N/A	Li et al.^34^	6–8 weeks	100.0	Brain	Microhemorrhages	N/R	N/R	0.0
	MAP2K1	N/A	Smits et al.^50^	P1	100.0	Brain	N/R	N/R	N/R	10.0
	MAP2K1	N/A	Sudduth et al.^51^	P1	N/R	All	N/R	N/R	N/R	0.0
	GPRASP1	N/A	Li et al.^21^	P3–P5	62.0	MCA	44.4%	Y	N/R	40.0
	BRAF+/−	N/A	Tu et al.^47^	6 weeks	100.0	Striatum, parietal cortex, cerebellum	14.3%	Y	Y	70.0
	BRAF−/−	N/A	Tu et al.^47^	6 weeks	100.0	Striatum, parietal cortex, cerebellum	Hemorrhages	N/R	Y	30.0
HHT1	Eng+/−	N/A	Satomi et al.^63^	Developmental	30.0	N/R	N/R	N/R	N/R	N/R
	Eng+/−	VEGF	Xu et al.^35^	8–10 weeks	88.9	Cortex or needle track	0.0%	N/R	N/R	100.0
	Eng−/−	Eng2fl/2fl;SM22a-Cre	Choi et al.^36^	Developmental	90.0	Various, spinal cord	Microhemorrhage	N/R	N/R	<50.0
	Eng−/−	VEGF	Choi et al.^36^	8–10 weeks	N/R	Injection site	Microhemorrhage	N/R	N/R	100.0
	Eng−/−	VEGF	Choi et al.^37^	8–10 weeks	N/R	Injection site	N/R	N/R	N/R	N/R
	Eng−/−	N/A	Han et al.^52^	P1–P3	0.0	Forebrain, cerebellum	N/R	N/R	N/R	40.0
	Eng−/−	N/A	Han et al.^52^	P8–P10	55.0	Forebrain, cerebellum	N/R	N/R	N/R	N/R
	Eng−/−	N/A	Han et al.^52^	P15–P17	86.0	Forebrain, cerebellum	N/R	N/R	N/R	N/R
	Eng−/−	VEGF stimulation, PdgfbicreER	Shabani et al.,^39^ Cells	8–10 weeks	N/R	N/R	Microhemorrhage	N/R	N/R	N/R
	Eng−/−	VEGF stimulation, PdgfbicreER	Shabani et al.,^38^ Biomedicines	8–10 weeks	N/R	N/R	Microhemorrhage	N/R	N/R	N/R
HHT2	Alk1+/−	VEGF	Chen et al.^40^	8 weeks	N/R	Injection site	Microhemorrhage	N/R	N/R	N/R
	Alk1+/−	VEGF	Hao et al.^41^	8–10 weeks	100.0	Injection site	N/R	N/R	N/R	N/R
	Alk1iECKO	VEGF	Chen et al.^17^	8 weeks	N/R	Injection site	Microhemorrhage	N/R	N/R	0.0
	Bmp10-iKO, Bmp9/10 dKO	N/A	Choi et al.^53^	P2–P3	100.0	N/R	Microhemorrhage	N/R	N/R	100.0
	Alk1−/−	VEGF	Choi et al.^37^	8–10 weeks	N/R	Injection site	N/R	N/R	N/R	N/R
	Alk1−/−	N/A	Han et al.^68^	N/R	58.0	Parieto-occipital, deep locations, frontal lobe	34.0%	N/R	N/R	35.0
	Alk1−/−	VEGF	Walker et al.^43^	8–10 weeks	100.0	Injection site	N/R	N/R	N/R	N/R
	Alk1−/−	VEGF	Walker et al.^44^	8–10 weeks	N/R	Injection site	N/R	N/R	N/R	N/R
	Alk1−/−	Flox-SM22Cre-Del	Milton et al.^64^	Developmental	100.0	Brain	Hemorrhages	N/R	N/R	4.5
	Alk1−/−	N/A	Park H et al.^49^	P4	N/R	N/R	N/R	N/R	N/R	0.0
	Alk1−/−	Bone marrow from Alk1 deleted mice into WT mice + AAV injection 4 weeks later + tamoxifen 2 weeks later	Shaligram et al.^45^	12 weeks	100.0	Basal ganglia	N/R	N/R	N/R	0.0–80.0
	Alk1−/−	VEGF stimulation, PdgfbicreER	Shabani et al.,^39^ Cells	8–10 weeks	N/R	N/R	Microhemorrhage	N/R	N/R	N/R
	Alk1−/−	VEGF stimulation	Shabani et al.,^39^ Cells	8–10 weeks	N/R	Right basal ganglia	Microhemorrhage	N/R	N/R	N/R
	Alk1KO	N/A	Scherschinski et al.^55^	P1	64.4	Right hemispheric striatum, left-hemispheric parietal cortex and midline cerebellum	3%+microhemorrhages	Y	N/R	97.0
	CRISPR/Cas9 system to delete Alk1	VEGF	Zhu et al.^46^	8 weeks	83.3	Injection site	N/R	N/R	N/R	N/R
	MGP−/−	N/A	Marin-Ramos et al.^65^	Developmental	100.0	N/R	N/R	N/R	N/R	45.0
	MGP−/−	N/A	Yao Y et al.^66^	Developmental	100.0	Brain	Hemorrhages	N/R	N/R	N/R
	MGP−/−	N/A	Yao J et al.^67^	Developmental	100.0	Brain	N/R	N/R	N/R	N/R
	Smad4−/−	N/A	Kim Y et al.^56^	P1	N/R	Hippocampus	N/R	N/R	N/R	Low
NOTCH	Tie2-tTA;TRE-Notch1	N/A	Murphy et al.^57^	P1	100.0	Brain	N/R	N/R	N/R	N/R
	Tie2-tTA;TRE-Notch4	N/A	Kim TN et al.^58^	P1	N/R	Brain	N/R	N/R	N/R	N/R
	Tie2-tTA;TRE-Notch4	N/A	Murphy et al.^57^	P1	N/R	Brain	N/R	N/R	N/R	N/R
	Tie2-tTA;TRE-Notch4	N/A	Murphy et al.^59^	P1	100.0	Cerebellum, neocortex	100.0%	N/R	Y	0.0
	Tie2-tTA;TRE-Notch4	N/A	Murphy et al.^25^	P1	N/R	Brain	N/R	N/R	N/R	100.0
	Tie2-tTa;TRE-Notch4	N/A	Huang et al.^60^	P1	100.0	N/R	N/R	Y	N/R	0.0
	Notch4	N/A	Nielsen et al.^69^	N/R	100.0	Especially cerebellum	100.0%	N/R	N/R	0.0
	Shh	N/A	Giarretta et al.^26^	250g	100.0	Injection site	N/R	N/R	N/R	100.0
	Rbpj−/−	N/A	Nielsen et al.^61^	P1	100.0	Brain	Hemorrhages	N/R	N/R	10.0
	Rbpj−/−	N/A	Adhicary et al.^27^	P1–P2	100.0	N/R	N/R	Y	N/R	N/R
	Rbpj−/−	N/A	Chapman et al.^62^	P1–P2	100.0	Cerebellum	N/R	N/R	N/R	N/R

N/A: not applicable; N/R: not reported; ibEC: induced in brain endothelial cells; MCA: medial cerebral artery; fl: floxed; KO: knocked-out; iECKO: induced knock-out in endothelial cells; WT: wild type; AAV: adeno-associated virus.

### Inclusions

The electronic search retrieved 643 records from PubMed and 1651 records from Embase (Supplemental Material; PRISMA flow chart). After excluding duplicates, 1935 studies were screened based on their titles and abstracts, resulting in 43 included studies. Two studies were removed after image duplication assessment, which led to a total of 41 articles to be included in the final analysis.

### Model characteristics

All studies used mice as target animals, except for one, which used rats.^26^ Young adult/adult animals were utilized in 16/41 of the studies.^26,33–47^ In mice, age over 6 weeks and in rats, weight around 250g was considered as adult animal, though this was not specified in the studies included. BAVMs were induced in mice pups in 18/41 of the studies^48–62^ and 6/41 of the studies used developmental models,^36,63–67^ where germ-line mutation was causative for bAVM formation. Two studies did not provide the model induction age.^68,69^ The most used mouse strain was C57BL/6. The models are presented in Table 2.

### Models

#### MAPK signaling related models

In eight articles MAPK signaling related models were investigated. The bAVMs were seen in the brain cortex (four articles), in basal ganglia area (three articles) or in cerebellum (two articles), and the models’ penetrance varied between 10.0% and 100.0% (median 100.0%) (from seven articles). BAVM rupture rate for MAPK signaling related models was assessed in four articles and meta-analysis revealed a significant increase in rupture rate in MAPK models compared to healthy controls (OR 8.90 [CI 1.23–64.14, I2 8.25%] (*n* = 3)). One armed MA showed an event rate of 9.2% [1.7%–36.0%] (*n* = 5). MA revealed no changes in survival between the MAPK models and the control group (OR 0.03 [CI 0.006–0.158, I2 43.42%] (*n* = 7)). One armed MA revealed 50.0% survival [20.0%–79.0%] (*n* = 7).

BAVM growth was reported in two articles^21,47^ and epileptic seizures were reported in one article^47^ (Tables 2–4).

**Table 4.** Summary of the animal models’ outcome measures.

Pathway	Survival			Rupture		
	Number of studies analyzed	OR [95% CI]	Effect	Number of studies analyzed	OR [95% CI]	Effect
2-arm model						
MAPK	7	0.03 [0.006–0.158], I2 43.42%	↓	3	8.90 [1.23–64.14], I2 8.25%	↑
HHT1	1	1.00 [0.018–55.80], I2 –	—	1	1.00 [0.02–55.80], I2–	—
HHT2	2	0.002 [0.000–0.018], I2–	—	1	3.19 [0.15–69.45], I2–	—
Notch	3	0.000 [0.000–0.018], I2 71.08%	↓	—	—	—
Pathway	Survival			Rupture		
	Number of studies analyzed	Event rate [95% CI]	Effect	Number of studies analyzed	Event rate [95% CI]	Effect
1-arm model						
MAPK	7	0.497 [0.207–0.789], I2 84.193%	↓	5	0.092 [0.017–0.366], I2 84.27%	↑
HHT1	2	0.719 [0.083–0.986], I2 80.877%	↓	1	0.050 [0.003–0.475], I2−	—
HHT2	7	0.243 [0.067–0.589], I2 88.359%	↓	2	0.218 [0.139–0.325], I2−	—
Notch	4	0.108 [0.009–0.606], I2 80.066%	↓	—	—	—

#### TGFbeta signaling related models

##### Genetic models related to HHT1

Seven articles used Eng mutation in their mouse models. BAVM development was mostly induced with VEGF (five articles). The penetrance of bAVMs was 0.0%–90.0% (median 70.5%) (from four articles). BAVMs were seen at the injection site (three articles), forebrain (two articles), cerebellum (one article) and in spinal cord (one article). BAVM rupture rate for models related to HHT1 was assessed in one study which showed no increased risk of rupture in HHT1 related models compared to healthy controls (OR 1.00 [CI 0.02–55.80, I2−]). One armed MA showed an event rate of 5.0% [0.3%–45.7%] (*n* = 1). Survival in HHT1 models was similar in study animals and control animals (OR 1.00 [CI 0.018–55.80, I2−]) (*n* = 1). One armed MA revealed 71.9% survival [8.3%–98.6%] (*n* = 2). Growth of the bAVMs or seizures were not reported (Tables 2–4).

##### Genetic models related to HHT2

Nineteen articles used HHT2 related mutation in their mouse models. Most of the articles (10/19) utilized external stimulation, usually VEGF, to induce bAVM development. The reported penetrance was 58.0%–100.0% (median 100.0%) (from 11 studies). BAVMs were seen mostly at the injection site (seven articles), but also parieto-occipital (two articles), cerebellar (one article) and deep locations (five articles) were possible. BAVM rupture rate for HHT2 related models was similar than in control animals (OR 3.19 [0.15–69.45, I2−]) (*n* = 1). One armed MA showed an event rate of 21.8% [CI 13.9–32.5] (*n* = 2). Survival in HHT2 models was similar than in control animals (OR 0.002 [CI 0.000–0.018, I2−]) (*n* = 2). One armed MA showed survival of 24.3% [CI 6.7–58.9] (*n* = 7). Growth of the bAVMs was reported in one article,^55^ and none of the publications reported on seizures (Tables 2–4).

#### Genetic models related to defective Notch signaling

Ten articles utilized defective Notch signaling in their bAVM models. No external stimulation was used to induce bAVM development. The model penetrance was 100.0% (median 100.0%) (from eight articles). BAVMs were seen in cerebellum (three articles), in cortex (one article) and at the injection site (one article). We were not able to calculate BAVM rupture rate for Notch signaling related models due to heterogenous reporting and lacking control data. Overall survival in Notch models was lower than in control animals (OR 0.000 [CI 0.000–0.018, I2 71.08%]) (*n* = 3). One armed MA showed survival of 10.8% [CI 0.9–60.6] (*n* = 4). BAVM growth was reported in two articles.^27,70^ One article^71^ reported seizures (Tables 2–4).

#### Image duplication assessment

We agreed on image-related issues in two articles: Cheng et al.^^72^^ and Ma et al.^73^ The issues were posted on PubPeer.com and the articles excluded from the data extraction and risk of bias assessment phase, which led to a total of 41 articles to be included in the final analysis.

#### Reporting quality and risk of bias assessment

A minority of the included studies reported on whether or not randomization (22.2%), blinding (42.2%), sample size calculation (11.1%) or preregistration of their research protocol (6.7%) had taken place (Figure 2). A majority of the studies (88.9%) included a conflict of interest statement (Figure 2).

Many details to assess risk of bias of the included studies were not reported. As a consequence many domains had an unclear risk of bias (Figure 3). For some studies, the questions related to assess the risk of selection bias were not applicable due to the nature of the animal model: inbreeding and cross-breeding resulted in whether animals had the specific mutation, and thus randomized and blinded allocation of the animals to the group were not applicable. A high risk of performance bias was seen in many studies (28.9%) because animals with bAVMs showed characteristics of having the disease (i.e. subarachnoid hemorrhage resulting in neurological deficits). A high risk of attrition bias was present in many studies (28.9%) as well, as not all animals described in the methods section were reported on in the results section, and no explanation for missing animals was provided. A detailed overview of the risk of bias assessment can be found in Figure 3.

## Discussion

We performed a systematic review of the existing literature to identify what animal models have been developed to study bAVMs and how their characteristics vary across models. We examined if animal models emulate the most important symptoms of human bAVMs through the following outcome parameters: bAVM rupture rate, survival, bAVM growth and epileptic seizures.

### General observations

Most bAVM models represented in the literature are with HHT related genetic background. Since only 2%–3% of human bAVMs appear with HHT syndrome,^74^ the animal model distribution seems biased. Models based on activating KRAS mutations, or other means of inducing overactivation of the MAPK signaling, seem more relevant for most human patients with sporadic bAVMs.

Most of the animal models represented lack in quality of reporting. This causes serious challenges when interpreting the results and overall hampers the models’ clinical relevance. Control data is often not reported. The clinically relevant outcome parameters of bAVM rupture rate and animal survival were reported often with high variability, even within the same model, indicating that external factors or a certain timeframe might affect the outcomes. Many of the models in this review present with high mortality for other reasons than full intracranial hemorrhages. This limits the model usability when interested in assessing more clinically relevant outcomes such as the presence of seizures or bAVM growth over time. In addition, models scarcely reported on bAVM growth or epileptic seizures.

### The models

KRAS mouse models were intriguing, since somatic KRAS mutations explain most sporadic bAVMs.^18^ When KRAS G12D models were induced in adult animals, it resulted in lower rupture rates than when they were induced in mice pups. This is similar to humans^4,33^ and could suggest that bAVMs induced by KRAS mutations are not necessarily congenital but may develop over postnatal life in patients. This concept is supported by the observation that children and young adults may develop de novo bAVMs after complete surgical removal of a bAVM.^75^ A significant proportion of patients are diagnosed with a bAVM due to subsequent ICH, but yet not all bAVMs rupture. Only one model utilized KRAS mutation G12V, which is generally considered to be the more aggressive one of the mutations.^76^ Induction of KRAS mutation specifically in brain endothelial cells caused a bAVM rupture rate similar to what is seen in human patients annually.^4,33^ This could imply that the bAVMs induced in mice by inserting an activating KRAS mutation in the brain endothelial cells, model the clinical course of human bAVMs rather accurately. One model utilized KRAS mutation G12V, which is generally considered to be the more aggressive one of the mutations,^76^ but unfortunately the study did not report on the disease’s clinical course in mice. Overall, bAVM growth or seizures were not reported in the KRAS models.

In addition to the bAVM model based on activating KRAS mutations, others developed a model that causes MAPK overactivation with an inducible HRAS mutation in endothelial cells.^34^ The survival of the mice was low, even though no full intracranial hemorrhages were reported. This indicates that these mice died due to other causes of death than stroke, which is relevant when assessing the usability of the model in further studies. Induction of bAVMs with MAP2K1 mutation and GPRASP1 and BRAF mutations raises the question whether other MAPK related mutations are also present in bAVMs, and whether a larger bAVM population than just KRAS mutated bAVMs could be treated with MAPK inhibitors.^76^ The reported bAVM rupture rates were higher than with the KRAS models (e.g., 44.4% with GPRASP1 mutation model vs. 0%–3.8%).

The most studied bAVM animal models utilized Eng and Alk1 mutations. With both genetic backgrounds, homozygous mutation is more effective to induce bAVMs. Also, in these models VEGF induces the development of bAVMs. This is interesting, since according to the second-hit theory, genetic variations alone are not sufficient to trigger bAVM formation, but a yet unknown external factor is believed to induce angiogenesis through upregulation of molecules like VEGF.^77^ With Eng and Alk1 models, bAVMs often develop at the VEGF injection site, which implies a need for high local concentration of external stimulus to see bAVM appearance. This represents a significant distinction compared to, for instance, KRAS-related bAVM animal models. Additionally, mutations in SMAD4 and MGP have been investigated in several studies. The SMAD4 mutation results in a rapidly progressing model, which does not accurately reflect human bAVM cases. Furthermore, MGP mutations have not been identified in human bAVM samples.

The Notch signaling related models included in this review lack information on all this review’s outcome parameters. The Notch4 model has been repeated in a quite large number of mice, and it causes a severe disease with high mortality between P20 and P36 due to intracranial hemorrhages.

### Relevance of current bAVM models for preclinical drug testing

Medical therapies for bAVMs are being developed and tested. Possibly the most intriguing target is the MAPK signaling pathway. MAPK inhibitors are already in clinical use with cancers, such as melanoma.^78^ Currently a phase II clinical trial is recruiting extracranial AVM patients into MEK inhibitor therapy.^79^ In extracranial vascular malformations clinical trials of using mTOR inhibitors have been conducted and sirolimus has become a new therapeutic option in recent years.^80^ Also, the recent discovery of active COX2 signaling behind inflammation mediated vascular remodeling in most bAVMs (Figure 1) offers an interesting target for medical therapies with COX2 inhibitors, such as celecoxib.^81^ With the arrival of potential drug therapy to downgrade or stabilize bAVMs, experimental models of bAVMs are increasingly needed for preclinical drug testing.

The purpose of utilizing animal models is to develop suitable therapies for human patients. In this systematic review we have identified many different bAVM animal models, which have limited clinical usability. For drug testing, it is important that the bAVM appearance and timing of natural course is standard. It is worth mentioning that in humans the mortality for bAVM is not 100% – even when untreated, but rather between 4% and 25% depending on the rupture status and treatment strategy.^82,83^ Therefore, the ideal bAVM animal model should not cause unreasonably high mortality.

### Limitations of the review

This study has several important limitations. The studies used rodent models (mice or rats) which may lack generalizability to human subjects. Animal models that do not generate bAVMs might not be published, which might generate publication bias. The risk of bias assessment showed poorly reported methodologies for every type of bAVM animal model, which might increase the risk of bias in the studies. We recommend future studies to report accordingly to guidelines, such as the ARRIVE guidelines.

## Conclusions

The models presented in this systematic review were heterogenous, and the reported data did not allow further analyses comparing the clinical course of bAVMs induced with different molecular pathology. While ruptures occurred in some of the models, epileptic seizures and bAVM growth were reported scarcely. The HHT related models were over-represented when compared to incidence in humans. Future models should prioritize standardization and comprehensive reporting to enhance their utility in preclinical drug development.

## Supplemental Material

sj-docx-1-jcb-10.1177_0271678X251409038 – Supplemental material for Translational relevance of animal models available on brain arteriovenous malformations, a systematic review Supplemental material, sj-docx-1-jcb-10.1177_0271678X251409038 for Translational relevance of animal models available on brain arteriovenous malformations, a systematic review by Sara Keränen, René Aquarius, Juhana Frösen, Carlijn R Hooijmans and Hieronymus D Boogaarts in Journal of Cerebral Blood Flow & Metabolism

sj-docx-2-jcb-10.1177_0271678X251409038 – Supplemental material for Translational relevance of animal models available on brain arteriovenous malformations, a systematic review Supplemental material, sj-docx-2-jcb-10.1177_0271678X251409038 for Translational relevance of animal models available on brain arteriovenous malformations, a systematic review by Sara Keränen, René Aquarius, Juhana Frösen, Carlijn R Hooijmans and Hieronymus D Boogaarts in Journal of Cerebral Blood Flow & Metabolism

sj-docx-3-jcb-10.1177_0271678X251409038 – Supplemental material for Translational relevance of animal models available on brain arteriovenous malformations, a systematic review Supplemental material, sj-docx-3-jcb-10.1177_0271678X251409038 for Translational relevance of animal models available on brain arteriovenous malformations, a systematic review by Sara Keränen, René Aquarius, Juhana Frösen, Carlijn R Hooijmans and Hieronymus D Boogaarts in Journal of Cerebral Blood Flow & Metabolism

sj-docx-4-jcb-10.1177_0271678X251409038 – Supplemental material for Translational relevance of animal models available on brain arteriovenous malformations, a systematic review Supplemental material, sj-docx-4-jcb-10.1177_0271678X251409038 for Translational relevance of animal models available on brain arteriovenous malformations, a systematic review by Sara Keränen, René Aquarius, Juhana Frösen, Carlijn R Hooijmans and Hieronymus D Boogaarts in Journal of Cerebral Blood Flow & Metabolism

sj-pdf-5-jcb-10.1177_0271678X251409038 – Supplemental material for Translational relevance of animal models available on brain arteriovenous malformations, a systematic review Supplemental material, sj-pdf-5-jcb-10.1177_0271678X251409038 for Translational relevance of animal models available on brain arteriovenous malformations, a systematic review by Sara Keränen, René Aquarius, Juhana Frösen, Carlijn R Hooijmans and Hieronymus D Boogaarts in Journal of Cerebral Blood Flow & Metabolism
