# Supplementary material for: Translational relevance of animal models available on brain arteriovenous malformations, a systematic review
Source: J Cereb Blood Flow Metab. 2026 Feb 24:0271678X251409038. Online ahead of print. doi: 10.1177/0271678X251409038 (PMC12935589; doi:10.1177/0271678X251409038)
Supplement: sj-docx-2-jcb-10.1177_0271678X251409038 – Supplemental material for Translational relevance of animal models available on brain arteriovenous malformations, a systematic review [file sj-docx-2-jcb-10.1177_0271678X251409038.docx]

**Identification of studies via databases and registers**

Records removed *before screening*:

Duplicate records removed (n = 359)

Records marked as ineligible by automation tools (n = 0)

Records removed for other reasons (n = 0)

Records identified from*:

Embase (n = 1651)

Pubmed (n = 643)

**Identification**

Records screened

(n = 1935)

Records excluded**

(n = 317)

Reports sought for retrieval

(n = 1618)

Reports not retrieved

(n = 8)

**Screening**

Reports excluded:

Reason 1: Not an original full length paper (n = 354)

Reason 2: Not an in-vivo animal study (n = 563)

Reason 3: No induction of bAVM (n = 648)

Reason 4: No appropriate control group (n = 0)

Reason 5: No histological/anatomical assessment of the bAVM (n = 4)

Reports assessed for eligibility

(n = 1610)

Studies included in review

(n = 41)

**Included**

*Consider, if feasible to do so, reporting the number of records identified from each database or register searched (rather than the total number across all databases/registers).

**If automation tools were used, indicate how many records were excluded by a human and how many were excluded by automation tools.

*From:*  Page MJ, McKenzie JE, Bossuyt PM, Boutron I, Hoffmann TC, Mulrow CD, et al. The PRISMA 2020 statement: an updated guideline for reporting systematic reviews. BMJ 2021;372:n71. doi: 10.1136/bmj.n71

For more information, visit: <http://www.prisma-statement.org/>
