# Supplementary material for: Translational relevance of animal models available on brain arteriovenous malformations, a systematic review
Source: J Cereb Blood Flow Metab. 2026 Feb 24:0271678X251409038. Online ahead of print. doi: 10.1177/0271678X251409038 (PMC12935589; doi:10.1177/0271678X251409038)
Supplement: sj-docx-4-jcb-10.1177_0271678X251409038 – Supplemental material for Translational relevance of animal models available on brain arteriovenous malformations, a systematic review [file sj-docx-4-jcb-10.1177_0271678X251409038.docx]

Supplemental Material

Keranen S et al.

Methods

*Pubmed search AVM component*

"Intracranial Arteriovenous Malformation*"[tiab] OR "Intracranial Arteriovenous Malformations"[MeSH Terms] OR "Brain AVM"[tiab] OR "bAVM"[tiab] OR ”Cerebral Arteriovenous Malformation*”[tiab] OR "Arteriovenous Aneurysm*"[tiab] OR "Galen Malformation*"[tiab] OR "Galen Vein Aneurysm*"[tiab] OR "Galenic Arteriovenous Malformation*"[tiab] OR "Vein of Galen Aneurysm*"[tiab] OR ”Aneurysm of the Vein of Galen”[tiab] OR ”Aneurysms of the Vein of Galen”[tiab] OR ”Great Brain Vein Aneurysm*”[tiab] OR ”Malformation of the Vein of Galen”[tiab] OR ”Malformations of the Vein of Galen”[tiab] OR ”Vein of Galen Arteriovenous Malformation*”[tiab] OR ”VGAM”[tiab] OR ”VGM”[tiab] OR (”Arteriovenous Malformation”[tiab] AND Brain[tiab]) OR ”Teleangiectasia, Hereditary Hemorrhagic”[tiab] OR ”Teleangiectasia, Hereditary Haemorrhagic”[tiab] OR ”Telangiectasia, Hereditary Hemorrhagic”[tiab] OR ”Telangiectasia, Hereditary Haemorrhagic”[tiab] OR ”Teleangiectasis, Hereditary Hemorrhagic”[tiab] OR ”Teleangiectasis, Hereditary Haemorrhagic”[tiab] OR ”Telangiectasis, Hereditary Hemorrhagic”[tiab] OR ”Telangiectasis, Hereditary Haemorrhagic”[tiab] OR ”Hereditary Hemorrhagic Teleangiectasia”[tiab] OR ”Hereditary Hemorrhagic Teleangiectasis”[tiab] OR ”Hereditary Hemorrhagic Telangiectasia”[tiab] OR ”Hereditary Hemorrhagic Telangiectasis”[tiab] OR ”Hereditary Haemorrhagic Teleangiectasia”[tiab] OR ”Hereditary Haemorrhagic Teleangiectasis”[tiab] OR ”Hereditary Haemorrhagic Telangiectasia”[tiab] OR ”Hereditary Haemorrhagic Telangiectasis”[tiab] OR ”Rendu Osler Weber Disease”[tiab] OR ”Rendu Osler Weber Syndrome”[tiab] OR ”Rendu Weber Osler Disease”[tiab] OR ”Rendu Weber Osler Syndrome”[tiab] OR ”Osler Weber Rendu Disease”[tiab] OR ”Osler Weber Rendu Syndrome”[tiab] OR ”Osler Disease”[tiab] OR ”Osler Syndrome”[tiab] OR ”Weber Osler Syndrome”[tiab] OR ”Osler Rendu Disease”[tiab] OR ”Weber Osler Disease”[tiab] OR ”Osler Rendu Weber Disease”[tiab] OR ”Osler Rendu Weber Syndrome”[tiab] OR ”Hereditary Hemorrhagic Angiomatosis”[tiab] OR ”Hereditary Haemorrhagic Angiomatosis”[tiab] OR ”Osler Rendu Syndrome”[tiab] OR ”Weber Rendu Osler Disease”[tiab] OR ”Weber Rendu Osler Syndrome”[tiab] OR ”Weber Osler Rendu Disease”[tiab] OR ”Weber Osler Rendu Syndrome”[tiab] OR ”Rendu Osler Angiomatosis”[tiab] OR ”Rendu Osler Disease”[tiab] OR ”Rendu Osler Syndrome”[tiab] OR ”Rendu-Osler-Weber's disease”[tiab] OR ”Teleangiectasia Hereditaria Haemorrhagica”[tiab]

*SYRCLE’s animal filter for Pubmed*

(animal experimentation[MeSH] OR models, animal[MeSH] OR Animals[Mesh:noexp] OR animal population groups [MeSH] OR chordata[MeSH Terms:noexp] OR vertebrates[MeSH Terms:noexp] OR amphibians[MeSH] OR birds[MeSH] OR fishes[MeSH] OR reptiles[MeSH] OR mammals[MeSH Terms:noexp] OR primates[MeSH Terms:noexp] OR eutheria[MeSH Terms:noexp] OR artiodactyla[MeSH] OR carnivore[MeSH] OR cephalopoda[MeSH] OR cetacea[MeSH] OR chiroptera[MeSH] OR elephants[MeSH] OR hyraxes[MeSH] OR insectivora[MeSH] OR lagomorpha[MeSH] OR marsupialia[MeSH] OR monotremata[MeSH] OR perissodactyla[MeSH] OR Proboscidea Mammal[MeSH Terms:noexp] OR rodentia[MeSH] OR scandentia[MeSH] OR sirenia[MeSH] OR cingulata[MeSH] OR haplorhini[MeSH Terms:noexp] OR strepsirhini[MeSH] OR platyrrhini[MeSH] OR tarsii[MeSH] OR catarrhini[MeSH Terms:noexp] OR cercopithecidae[MeSH] OR hylobatidae[MeSH] OR hominidae[MeSH Terms:noexp] OR gorilla gorilla[MeSH] OR pan paniscus[MeSH] OR pan troglodytes[MeSH] OR pongo[MeSH]) OR ((rat[tiab] OR rats[tiab] OR animal[tiab] OR animals[tiab] OR mice[tiab] OR in vivo[tiab] OR mouse[tiab] OR rabbit[tiab] OR rabbits[tiab] OR murine[tiab] OR pig[tiab] OR pigs[tiab] OR dog[tiab] OR dogs[tiab] OR bovine[tiab] OR fish[tiab] OR vertebrate[tiab] OR vertebrates[tiab] OR cat[tiab] OR cats[tiab] OR rodent[tiab] OR rodents[tiab] OR mammal[tiab] OR mammals[tiab] OR chicken[tiab] OR chickens[tiab] OR monkey[tiab] OR monkeys[tiab] OR sheep[tiab] OR canine[tiab] OR canines[tiab] OR porcine[tiab] OR cattle[tiab] OR bird[tiab] OR birds[tiab] OR hamster[tiab] OR hamsters[tiab] OR primate[tiab] OR primates[tiab] OR cow[tiab] OR cows[tiab] OR chick[tiab] OR horse[tiab] OR horses[tiab] OR avian[tiab] OR avians[tiab] OR calf[tiab] OR swine[tiab] OR swines[tiab] OR xenopus[tiab] OR turkeys[tiab] OR bear[tiab] OR bears[tiab] OR frog[tiab] OR frogs[tiab] OR zebrafish[tiab] OR goat[tiab] OR goats[tiab] OR equine[tiab] OR calves[tiab] OR poultry[tiab] OR macaque[tiab] OR macaques[tiab] OR mole[tiab] OR moles[tiab] OR ovine[tiab] OR lamb[tiab] OR lambs[tiab] OR fishes[tiab] OR diptera[tiab] OR amphibian[tiab] OR amphibians[tiab] OR snake[tiab] OR snakes[tiab] OR ruminant[tiab] OR ruminants[tiab] OR hen[tiab] OR hens[tiab] OR piglet[tiab] OR piglets[tiab] OR feline[tiab] OR felines[tiab] OR simian[tiab] OR simians[tiab] OR laevis[tiab] OR trout[tiab] OR trouts[tiab] OR teleost[tiab] OR teleosts[tiab] OR salmon[tiab] OR salmons[tiab] OR seal[tiab] OR seals[tiab] OR bull[tiab] OR bulls[tiab] OR ewe[tiab] OR ewes[tiab] OR hedgehog[tiab] OR hedgehogs[tiab] OR macaca[tiab] OR macacas[tiab] OR proteus[tiab] OR pigeon[tiab] OR pigeons[tiab] OR bat[tiab] OR bats[tiab] OR duck[tiab] OR ducks[tiab] OR chimpanzee[tiab] OR chimpanzees[tiab] OR baboon[tiab] OR baboons[tiab] OR deer[tiab] OR rana[tiab] OR ranas[tiab] OR carp[tiab] OR carps[tiab] OR heifer[tiab] OR swallow[tiab] OR swallows[tiab] OR lizard[tiab] OR lizards[tiab] OR canis[tiab] OR sow[tiab] OR sows[tiab] OR cynomolgus[tiab] OR quail[tiab] OR quails[tiab] OR reptile[tiab] OR reptiles[tiab] OR turtle[tiab] OR turtles[tiab] OR buffalo[tiab] OR gerbil[tiab] OR gerbils[tiab] OR boar[tiab] OR boars[tiab] OR squirrel[tiab] OR squirrels[tiab] OR oncorhynchus[tiab] OR mus[tiab] OR toad[tiab] OR toads[tiab] OR fowl[tiab] OR fowls[tiab] OR rerio[tiab] OR danio[tiab] OR ara[tiab] OR aras[tiab] OR musculus[tiab] OR tadpole[tiab] OR tadpoles[tiab] OR mulatta[tiab] OR salmo[tiab] OR ram[tiab] OR eagle[tiab] OR eagles[tiab] OR ferret[tiab] OR ferrets[tiab] OR goldfish[tiab] OR catfish[tiab] OR whale[tiab] OR whales[tiab] OR fox[tiab] OR foxes[tiab] OR ape[tiab] OR apes[tiab] OR elephant[tiab] OR elephants[tiab] OR bos[tiab] OR marmoset[tiab] OR marmosets[tiab] OR cod[tiab] OR cods[tiab] OR shark[tiab] OR sharks[tiab] OR wolf[tiab] OR eel[tiab] OR eels[tiab] OR auratus[tiab] OR rattus[tiab] OR zebra[tiab] OR zebras[tiab] OR tilapia[tiab] OR tilapias[tiab] OR gilt[tiab] OR camel[tiab] OR camels[tiab] OR squid[tiab] OR gallus[tiab] OR marsupial[tiab] OR marsupials[tiab] OR vole[tiab] OR voles[tiab] OR fascicularis[tiab] OR ovis[tiab] OR salmonid[tiab] OR salmonids[tiab] OR tiger[tiab] OR tigers[tiab] OR dolphin[tiab] OR dolphins[tiab] OR robin[tiab] OR robins[tiab] OR carpio[tiab] OR opossum[tiab] OR opossums[tiab] OR cyprinus[tiab] OR salamander[tiab] OR salamanders[tiab] OR felis[tiab] OR mink[tiab] OR minks[tiab] OR swan[tiab] OR swans[tiab] OR norvegicus[tiab] OR bufo[tiab] OR torpedo[tiab] OR bass[tiab] OR lamprey[tiab] OR lampreys[tiab] OR sus[tiab] OR python[tiab] OR pythons[tiab] OR tetrapod[tiab] OR tetrapods[tiab] OR shrew[tiab] OR shrews[tiab] OR lion[tiab] OR lions[tiab] OR hog[tiab] OR hogs[tiab] OR songbird[tiab] OR songbirds[tiab] OR oreochromis[tiab] OR starling[tiab] OR starlings[tiab] OR caprine[tiab] OR carassius[tiab] OR owl[tiab] OR owls[tiab] OR newt[tiab] OR newts[tiab] OR papio[tiab] OR scrofa[tiab] OR hare[tiab] OR hares[tiab] OR gorilla[tiab] OR gorillas[tiab] OR flounder[tiab] OR flounders[tiab] OR goose[tiab] OR herring[tiab] OR herrings[tiab] OR therian[tiab] OR buffaloes[tiab] OR canary[tiab] OR sparrow[tiab] OR sparrows[tiab] OR microtus[tiab] OR octopus[tiab] OR troglodytes[tiab] OR tuna[tiab] OR amphibia[tiab] OR chinchilla[tiab] OR chinchillas[tiab] OR ide[tiab] OR oryzias[tiab] OR cervus[tiab] OR kangaroo[tiab] OR kangaroos[tiab] OR armadillo[tiab] OR armadillos[tiab] OR callithrix[tiab] OR pan troglodytes[tiab] OR saimiri[tiab] OR cichlid[tiab] OR cichlids[tiab] OR donkey[tiab] OR donkeys[tiab] OR bream[tiab] OR char[tiab] OR chars[tiab] OR finch[tiab] OR raccoon[tiab] OR raccoons[tiab] OR bothrops[tiab] OR anguilla[tiab] OR perch[tiab] OR cricetus[tiab] OR seabird[tiab] OR seabirds[tiab] OR buck[tiab] OR bucks[tiab] OR naja[tiab] OR coturnix[tiab] OR salmonids[tiab] OR geese[tiab] OR minnow[tiab] OR minnows[tiab] OR raptor[tiab] OR raptors[tiab] OR merione[tiab] OR meriones[tiab] OR rodentia[tiab] OR elaphus[tiab] OR amniote[tiab] OR amniotes[tiab] OR elasmobranch[tiab] OR emu[tiab] OR emus[tiab] OR peromyscus[tiab] OR hominid[tiab] OR hominids[tiab] OR bubalus[tiab] OR crotalus[tiab] OR gull[tiab] OR gulls[tiab] OR anas[tiab] OR anura[tiab] OR lemur[tiab] OR lemurs[tiab] OR crow[tiab] OR crows[tiab] OR camelus[tiab] OR gibbon[tiab] OR gibbons[tiab] OR waterfowl[tiab] OR parrot[tiab] OR parrots[tiab] OR eels[tiab] OR cob[tiab] OR stickleback[tiab] OR sticklebacks[tiab] OR columba[tiab] OR mesocricetus[tiab] OR ambystoma[tiab] OR raven[tiab] OR ravens[tiab] OR gadus[tiab] OR penguin[tiab] OR penguins[tiab] OR orangutan[tiab] OR orangutans[tiab] OR sturgeon[tiab] OR sturgeons[tiab] OR cuniculus[tiab] OR aves[tiab] OR virginianus[tiab] OR cephalopod[tiab] OR cephalopods[tiab] OR cebus[tiab] OR sparus[tiab] OR tortoise[tiab] OR tortoises[tiab] OR guttata[tiab] OR morhua[tiab] OR unguiculatus[tiab] OR dogfish[tiab] OR vulpes[tiab] OR mallard[tiab] OR mallards[tiab] OR apodemus[tiab] OR alligator[tiab] OR alligators[tiab] OR oryctolagus[tiab] OR llama[tiab] OR llamas[tiab] OR reindeer[tiab] OR mustela[tiab] OR duckling[tiab] OR ducklings[tiab] OR wolves[tiab] OR sander[tiab] OR amazona[tiab] OR zebu[tiab] OR badger[tiab] OR badgers[tiab] OR dove[tiab] OR doves[tiab] OR ictalurus[tiab] OR capra[tiab] OR capras[tiab] OR equus[tiab] OR camelid[tiab] OR camelids[tiab] OR poecilia[tiab] OR mule[tiab] OR mules[tiab] OR perciformes[tiab] OR salvelinus[tiab] OR labrax[tiab] OR cyprinidae[tiab] OR ariidae[tiab] OR crocodile[tiab] OR crocodiles[tiab] OR fundulus[tiab] OR dicentrarchus[tiab] OR clarias[tiab] OR cercopithecus[tiab] OR chiroptera[tiab] OR alpaca[tiab] OR alpacas[tiab] OR pike[tiab] OR pikes[tiab] OR paralichthys[tiab] OR puma[tiab] OR pumas[tiab] OR didelphis[tiab] OR pisces[tiab] OR macropus[tiab] OR triturus[tiab] OR bison[tiab] OR bisons[tiab] OR epinephelus[tiab] OR gasterosteus[tiab] OR panthera[tiab] OR acipenser[tiab] OR mackerel[tiab] OR mackerels[tiab] OR tamarin[tiab] OR tamarins[tiab] OR ostrich[tiab] OR anolis[tiab] OR vervet[tiab] OR vervets[tiab] OR wallaby[tiab] OR glareolus[tiab] OR beaver[tiab] OR beavers[tiab] OR dromedary[tiab] OR catus[tiab] OR killifish[tiab] OR pimephales[tiab] OR promelas[tiab] OR aotus[tiab] OR phoca[tiab] OR panda[tiab] OR pandas[tiab] OR porpoise[tiab] OR porpoises[tiab] OR myotis[tiab] OR yak[tiab] OR yaks[tiab] OR agkistrodon[tiab] OR vipera[tiab] OR otter[tiab] OR otters[tiab] OR turbot[tiab] OR turbots[tiab] OR squamate[tiab] OR carnivora[tiab] OR mullet[tiab] OR mullets[tiab] OR hawk[tiab] OR hawks[tiab] OR taeniopygia[tiab] OR seahorse[tiab] OR seahorses[tiab] OR poecilia reticulata[tiab] OR falcon[tiab] OR falcons[tiab] OR prosimian[tiab] OR prosimians[tiab] OR parus[tiab] OR perca[tiab] OR fingerling[tiab] OR fingerlings[tiab] OR antelope[tiab] OR antelopes[tiab] OR tupaia[tiab] OR passeriformes[tiab] OR sepia[tiab] OR saguinus[tiab] OR coyote[tiab] OR coyotes[tiab] OR pongo[tiab] OR meleagris[tiab] OR reptilia[tiab] OR lepus[tiab] OR psittacine[tiab] OR hagfish[tiab] OR warbler[tiab] OR warblers[tiab] OR russell's viper[tiab] OR russell's vipers[tiab] OR smolt[tiab] OR smolts[tiab] OR budgerigar[tiab] OR sardine[tiab] OR sardines[tiab] OR cavia[tiab] OR cavias[tiab] OR hyla[tiab] OR pleurodeles[tiab] OR siluriformes[tiab] OR great tit[tiab] OR great tits[tiab] OR guppy[tiab] OR bonobo[tiab] OR bonobos[tiab] OR rutilus[tiab] OR trichosurus[tiab] OR muridae[tiab] OR phodopus[tiab] OR channa[tiab] OR squalus[tiab] OR lynx[tiab] OR sturnus[tiab] OR petromyzon[tiab] OR vitulina[tiab] OR monodelphis[tiab] OR cuttlefish[tiab] OR adder[tiab] OR adders[tiab] OR lepomis[tiab] OR canaria[tiab] OR gambusia[tiab] OR guppies[tiab] OR xiphophorus[tiab] OR flatfish[tiab] OR koala[tiab] OR koalas[tiab] OR labeo[tiab] OR stingray[tiab] OR stingrays[tiab] OR chelonia[tiab] OR lampetra[tiab] OR spermophilus[tiab] OR crocodilian[tiab] OR passer domesticus[tiab] OR sciurus[tiab] OR artiodactyla[tiab] OR ranidae[tiab] OR corvus[tiab] OR necturus[tiab] OR platypus[tiab] OR canaries[tiab] OR bovid[tiab] OR lagopus[tiab] OR trimeresurus[tiab] OR gariepinus[tiab] OR marten[tiab] OR martens[tiab] OR drosophilidae[tiab] OR mugil[tiab] OR sunfish[tiab] OR porcellus[tiab] OR cypriniformes[tiab] OR alouatta[tiab] OR scophthalmus[tiab] OR anser[tiab] OR electrophorus[tiab] OR putorius[tiab] OR iguana[tiab] OR iguanas[tiab] OR lama[tiab] OR lamas[tiab] OR takifugu[tiab] OR circus[tiab] OR eptesicus[tiab] OR flycatcher[tiab] OR galago[tiab] OR galagos[tiab] OR trachemys[tiab] OR lungfish[tiab] OR characiformes[tiab] OR shorebird[tiab] OR shorebirds[tiab] OR giraffe[tiab] OR giraffes[tiab] OR micropterus[tiab] OR scyliorhinus[tiab] OR cichlidae[tiab] OR loligo[tiab] OR porcupine[tiab] OR porcupines[tiab] OR chub[tiab] OR chubs[tiab] OR solea[tiab] OR pleuronectes[tiab] OR hylidae[tiab] OR viperidae[tiab] OR echis[tiab] OR sorex[tiab] OR anchovy[tiab] OR lagomorph[tiab] OR ostriches[tiab] OR vulture[tiab] OR vultures[tiab] OR whitefish[tiab] OR araneus[tiab] OR jird[tiab] OR jirds[tiab] OR tern[tiab] OR esox[tiab] OR drake[tiab] OR drakes[tiab] OR elapidae[tiab] OR gallopavo[tiab] OR chordata[tiab] OR myodes[tiab] OR caretta[tiab] OR serinus[tiab] OR grouse[tiab] OR misgurnus[tiab] OR meles[tiab] OR blackbird[tiab] OR blackbirds[tiab] OR coregonus[tiab] OR bobwhite[tiab] OR bobwhites[tiab] OR heteropneustes[tiab] OR mammoth[tiab] OR mammoths[tiab] OR turdus[tiab] OR rhinella[tiab] OR ateles[tiab] OR characidae[tiab] OR clupea[tiab] OR bungarus [tiab] OR brill[tiab] OR struthio camelus[tiab] OR sloth[tiab] OR sloths[tiab] OR pteropus[tiab] OR sculpin[tiab] OR anthropoids[tiab] OR pollock[tiab] OR pollocks[tiab] OR morone[tiab] OR pan paniscus[tiab] OR litoria[tiab] OR chipmunk[tiab] OR chipmunks[tiab] OR balaenoptera[tiab] OR marmota[tiab] OR melopsittacus[tiab] OR hyrax[tiab] OR lemming[tiab] OR lemmings[tiab] OR halibut[tiab] OR hylobates[tiab] OR lates[tiab] OR caiman[tiab] OR caimans[tiab] OR sigmodon[tiab] OR stenella[tiab] OR barbel[tiab] OR barbels[tiab] OR sterna[tiab] OR parakeet[tiab] OR parakeets[tiab] OR phocoena[tiab] OR leptodactylus[tiab] OR canidae[tiab] OR buteo[tiab] OR harengus[tiab] OR gopher[tiab] OR gophers[tiab] OR marmot[tiab] OR marmots[tiab] OR gosling[tiab] OR goslings[tiab] OR platichthys[tiab] OR gar[tiab] OR gars[tiab] OR sebastes[tiab] OR marsupialia[tiab] OR notophthalmus[tiab] OR gazelle[tiab] OR gazelles[tiab] OR insectivora[tiab] OR paridae[tiab] OR felidae[tiab] OR russula[tiab] OR galliformes[tiab] OR bombina[tiab] OR colobus [tiab] OR echidna[tiab] OR echidnas[tiab] OR seabass[tiab] OR syncerus[tiab] OR plaice[tiab] OR blue tit[tiab] OR blue tits[tiab] OR pagrus[tiab] OR catfishes[tiab] OR cetacea[tiab] OR barbus[tiab] OR cygnus[tiab] OR ficedula[tiab] OR chamois[tiab] OR colubridae[tiab] OR perches[tiab] OR coelacanth[tiab] OR fitch[tiab] OR urodela[tiab] OR cynops[tiab] OR martes[tiab] OR halichoerus[tiab] OR aix[tiab] OR salmonidae[tiab] OR leuciscus[tiab] OR magpie[tiab] OR magpies[tiab] OR silurus[tiab] OR whiting[tiab] OR whitings[tiab] OR anseriformes[tiab] OR colinus[tiab] OR rhea[tiab] OR chlorocebus[tiab] OR octodon[tiab] OR acinonyx[tiab] OR mouflon[tiab] OR mouflons[tiab] OR ibex[tiab] OR tetraodon[tiab] OR bufonidae[tiab] OR equidae[tiab] OR jackal[tiab] OR cephalopoda[tiab] OR dendroaspis[tiab] OR glama[tiab] OR muskrat[tiab] OR muskrats[tiab] OR sable[tiab] OR sables[tiab] OR wildebeest[tiab] OR streptopelia[tiab] OR albifrons[tiab] OR vespertilionidae[tiab] OR woodpecker[tiab] OR woodpeckers[tiab] OR muntjac[tiab] OR muntjacs[tiab] OR archosaur[tiab] OR branta[tiab] OR cricetulus[tiab] OR megalobrama[tiab] OR poeciliidae[tiab] OR desmodus[tiab] OR snakehead[tiab] OR snakeheads[tiab] OR tench[tiab] OR teal[tiab] OR teals[tiab] OR bandicoot[tiab] OR bandicoots[tiab] OR apteronotus[tiab] OR phyllostomidae[tiab] OR crocidura[tiab] OR buzzard[tiab] OR buzzards[tiab] OR larimichthys[tiab] OR cercocebus[tiab] OR pipistrellus[tiab] OR erithacus[tiab] OR impala[tiab] OR impalas[tiab] OR rousettus[tiab] OR haddock[tiab] OR haddocks[tiab] OR tinca[tiab] OR ratite[tiab] OR calidris[tiab] OR cynoglossus[tiab] OR hypophthalmichthys[tiab] OR bullock[tiab] OR bullocks[tiab] OR dromedaries[tiab] OR alectoris[tiab] OR filly[tiab] OR salamandra[tiab] OR cingulata[tiab] OR bitis[tiab] OR grus[tiab] OR ammodytes[tiab] OR macaw[tiab] OR macaws[tiab] OR hypoleuca[tiab] OR sapajus[tiab] OR cyprinodontiformes[tiab] OR hippopotamus[tiab] OR pelophylax[tiab] OR capybara[tiab] OR capybaras[tiab] OR weasel[tiab] OR weasels[tiab] OR cairina[tiab] OR cynomys[tiab] OR lutra[tiab] OR cockatoo[tiab] OR cockatoos[tiab] OR lachesis[tiab] OR lagomorpha[tiab] OR rupicapra[tiab] OR daboia[tiab] OR orang utan[tiab] OR orang utans[tiab] OR platyrrhini[tiab] OR charadriiformes[tiab] OR micrurus[tiab] OR psittaciformes[tiab] OR spalax[tiab] OR loris[tiab] OR mustelidae[tiab] OR sylvilagus[tiab] OR vitticeps[tiab] OR cockatiel[tiab] OR mustelus[tiab] OR cottus[tiab] OR erythrocebus[tiab] OR dipodomys[tiab] OR platessa[tiab] OR callicebus[tiab] OR loricariidae[tiab] OR catostomus[tiab] OR cuneata[tiab] OR cyanistes[tiab] OR cyprinodon[tiab] OR sigmodontinae[tiab] OR elasmobranchii[tiab] OR trichechus[tiab] OR sauropsid[tiab] OR xenarthra[tiab] OR dormouse[tiab] OR perissodactyla[tiab] OR nautilus[tiab] OR cirrhinus[tiab] OR gulo[tiab] OR tragelaphus[tiab] OR merula[tiab] OR numida[tiab] OR sciaenidae[tiab] OR cerastes[tiab] OR sciuridae[tiab] OR gibbosus[tiab] OR octopuses[tiab] OR eland[tiab] OR elands[tiab] OR phyllomedusa[tiab] OR pogona[tiab] OR walrus[tiab] OR agamidae[tiab] OR leptodactylidae[tiab] OR ridibundus[tiab] OR leontopithecus[tiab] OR anteater[tiab] OR anteaters[tiab] OR pelodiscus[tiab] OR cebidae[tiab] OR columbianus[tiab] OR pelteobagrus fulvidraco[tiab] OR hominoidea[tiab] OR mandrillus[tiab] OR zonotrichia leucophrys[tiab] OR agama[tiab] OR gobiocypris[tiab] OR bearded dragon[tiab] OR bearded dragons[tiab] OR sarotherodon[tiab] OR talpa[tiab] OR discoglossus[tiab] OR hagfishes[tiab] OR sphenodon[tiab] OR gudgeon[tiab] OR amphiuma[tiab] OR aythya[tiab] OR tenrec[tiab] OR tenrec[tiab] OR hominidae[tiab] OR risoria[tiab] OR salamandridae[tiab] OR camelidae[tiab] OR columbiformes[tiab] OR latimeria[tiab] OR plover[tiab] OR plovers[tiab] OR afrotheria[tiab] OR falco sparverius[tiab] OR polecat[tiab] OR polecats[tiab] OR crotalinae[tiab] OR salvadora[tiab] OR tarsier[tiab] OR lucioperca[tiab] OR anchovies[tiab] OR lungfishes[tiab] OR terrapin[tiab] OR dromaius novaehollandiae[tiab] OR lateolabrax[tiab] OR eigenmannia[tiab] OR pelamis[tiab] OR theropithecus[tiab] OR murinae[tiab] OR gander[tiab] OR gymnotus[tiab] OR pseudacris[tiab] OR gymnophiona[tiab] OR gymnotiformes[tiab] OR laticauda[tiab] OR falconiformes[tiab] OR dugong[tiab] OR dugongs[tiab] OR pintail[tiab] OR pintails[tiab] OR rook[tiab] OR rooks[tiab] OR lasiurus[tiab] OR catshark[tiab] OR catsharks[tiab] OR micropogonias[tiab] OR red junglefowl[tiab] OR paddlefish[tiab] OR ophiophagus[tiab] OR hollandicus[tiab] OR nymphicus[tiab] OR pimelodidae[tiab] OR aepyceros[tiab] OR cobitidae[tiab] OR strigiformes[tiab] OR cobitis[tiab] OR dormice[tiab] OR alytes[tiab] OR calloselasma[tiab] OR guanaco[tiab] OR phasianidae[tiab] OR round goby[tiab] OR trichogaster[tiab] OR catarrhini[tiab] OR eelpout[tiab] OR eelpouts[tiab] OR galaxias[tiab] OR gaur[tiab] OR pungitius[tiab] OR suslik[tiab] OR susliks[tiab] OR flatfishes[tiab] OR percidae[tiab] OR caprinae[tiab] OR todarodes[tiab] OR osmerus[tiab] OR ameiurus[tiab] OR anthropoidea[tiab] OR castor canadensis[tiab] OR pouting[tiab] OR poutings[tiab] OR tetraodontiformes[tiab] OR arvicolinae[tiab] OR siamang[tiab] OR siamangs[tiab] OR castor fiber[tiab] OR nomascus[tiab] OR red knot[tiab] OR red knots[tiab] OR syngnathidae[tiab] OR iguanidae[tiab] OR eretmochelys[tiab] OR ursidae[tiab] OR callimico[tiab] OR columbidae[tiab] OR microhylidae[tiab] OR anaxyrus[tiab] OR menidia[tiab] OR pipistrelle[tiab] OR greylag[tiab] OR pipidae[tiab] OR scandentia[tiab] OR bowfin[tiab] OR bowfins[tiab] OR dendrobatidae[tiab] OR zenaida[tiab] OR bushbaby[tiab] OR harrier[tiab] OR harriers[tiab] OR macropodidae[tiab] OR pygerythrus[tiab] OR clupeidae[tiab] OR odorrana[tiab] OR corvidae[tiab] OR jerboa[tiab] OR jerboas[tiab] OR canutus[tiab] OR hylobatidae[tiab] OR clupeiformes[tiab] OR great cormorant[tiab] OR great cormorants[tiab] OR scorpaeniformes[tiab] OR chondrostean[tiab] OR garfish[tiab] OR proboscidea[tiab] OR psetta[tiab] OR diapsid[tiab] OR serotinus[tiab] OR tetrao[tiab] OR walruses[tiab] OR carcharhiniformes[tiab] OR leucoraja[tiab] OR pumpkinseed[tiab] OR dosidicus[tiab] OR acipenseriformes[tiab] OR daubentonii[tiab] OR emberizidae[tiab] OR gadiformes[tiab] OR hyraxes[tiab] OR stizostedion[tiab] OR wolverine[tiab] OR wolverines[tiab] OR lissotriton[tiab] OR acanthurus[tiab] OR centrarchidae[tiab] OR gloydius[tiab] OR laurasiatheria[tiab] OR limosa[tiab] OR psittacula[tiab] OR leporidae[tiab] OR proteidae[tiab] OR zander[tiab] OR zanders[tiab] OR arapaima[tiab] OR bagridae[tiab] OR cyprinodontidae[tiab] OR mithun[tiab] OR pandion[tiab] OR jackdaw[tiab] OR jackdaws[tiab] OR procyonidae[tiab] OR carus[tiab] OR jaculus[tiab] OR salmoniformes[tiab] OR common sole[tiab] OR common soles[tiab] OR protobothrops[tiab] OR calamita[tiab] OR brachyteles[tiab] OR trionyx[tiab] OR turdidae[tiab] OR boidae[tiab] OR luscinia[tiab] OR pugnax[tiab] OR euarchontoglires[tiab] OR saithe[tiab] OR saithes[tiab] OR symphalangus[tiab] OR aardvark[tiab] OR aardvarks[tiab] OR oystercatcher[tiab] OR oystercatchers[tiab] OR arius[tiab] OR corydoras[tiab] OR poacher[tiab] OR poachers[tiab] OR aurochs[tiab] OR cebuella[tiab] OR crecca[tiab] OR lemuridae[tiab] OR sirenia[tiab] OR lemmus[tiab] OR perdix[tiab] OR glires[tiab] OR lepidosaur[tiab] OR muskox[tiab] OR deinagkistrodon[tiab] OR pholidota[tiab] OR holocephali[tiab] OR cercopithecinae[tiab] OR clariidae[tiab] OR agapornis[tiab] OR doryteuthis[tiab] OR tyrannidae[tiab] OR dicroglossidae[tiab] OR godwit[tiab] OR godwits[tiab] OR monedula[tiab] OR pongidae[tiab] OR atheriniformes[tiab] OR colobinae[tiab] OR lophocebus[tiab] OR atelidae[tiab] OR cottidae[tiab] OR leucopsis[tiab] OR acanthuridae[tiab] OR didelphimorphia[tiab] OR elver[tiab] OR elvers[tiab] OR lapponica[tiab] OR dermoptera[tiab] OR european hake[tiab] OR european hakes[tiab] OR gerbillinae[tiab] OR banteng[tiab] OR hartebeest[tiab] OR hartebeests[tiab] OR hogget[tiab] OR haematopus[tiab] OR anguis fragilis[tiab] OR grey heron[tiab] OR grey herons[tiab] OR blue whiting[tiab] OR blue whitings[tiab] OR furnariidae[tiab] OR macrovipera[tiab] OR esocidae[tiab] OR lapwing[tiab] OR lapwings[tiab] OR mylopharyngodon[tiab] OR wallabia[tiab] OR beloniformes[tiab] OR potoroo[tiab] OR potoroos[tiab] OR athene noctua[tiab] OR pleuronectidae[tiab] OR bushbabies[tiab] OR muscicapidae[tiab] OR alligatoridae[tiab] OR fuligula[tiab] OR bush baby[tiab] OR guineafowl[tiab] OR spoonbill[tiab] OR spoonbills[tiab] OR viverridae[tiab] OR catostomidae[tiab] OR zebrafishes[tiab] OR ibexes[tiab] OR vendace[tiab] OR estrildidae[tiab] OR monotremata[tiab] OR sepiella[tiab] OR ambystomatidae[tiab] OR shelduck[tiab] OR shelducks[tiab] OR treeshrew[tiab] OR treeshrews[tiab] OR hoplobatrachus[tiab] OR pochard[tiab] OR hoolock[tiab] OR hoolocks[tiab] OR lynxes[tiab] OR antilope[tiab] OR antilopes[tiab] OR blackbuck[tiab] OR blackbucks[tiab] OR cricetinae[tiab] OR paramisgurnus[tiab] OR skylark[tiab] OR skylarks[tiab] OR soleidae[tiab] OR allobates[tiab] OR northern wheatear[tiab] OR northern wheatears[tiab] OR pitheciidae[tiab] OR takin[tiab] OR theria[tiab] OR vanellus[tiab] OR galaxiidae[tiab] OR lorisidae[tiab] OR ostralegus[tiab] OR palaeognathae[tiab] OR stone loach[tiab] OR alauda[tiab] OR callitrichinae[tiab] OR caniformia[tiab] OR duttaphrynus[tiab] OR ictaluridae[tiab] OR osteoglossiformes[tiab] OR poultries[tiab] OR curema[tiab] OR ruddy turnstone[tiab] OR ruddy turnstones[tiab] OR sheatfish[tiab] OR sunfishes[tiab] OR centropomidae[tiab] OR hemachatus[tiab] OR platalea[tiab] OR thamnophilidae[tiab] OR song thrush[tiab] OR atherinopsidae[tiab] OR siluridae[tiab] OR tadorna[tiab] OR chroicocephalus[tiab] OR ermine[tiab] OR ermines[tiab] OR gavialis[tiab] OR ruff[tiab] OR tupaiidae[tiab] OR diprotodontia[tiab] OR hyaenidae[tiab] OR antilopinae[tiab] OR crocodylidae[tiab] OR herpestidae[tiab] OR hippopotamidae[tiab] OR northern shoveler[tiab] OR round gobies[tiab] OR cheirogaleidae[tiab] OR indriidae[tiab] OR fundulidae[tiab] OR pythonidae[tiab] OR rhynchocephalia[tiab] OR anodorhynchus[tiab] OR red-backed shrike[tiab] OR red-backed shrikes[tiab] OR triakidae[tiab] OR phalangeridae[tiab] OR aoudad[tiab] OR boreoeutheria[tiab] OR eurasian jay[tiab] OR eurasian jays[tiab] OR feliformia[tiab] OR haplorhini[tiab] OR osteoglossidae[tiab] OR paenungulata[tiab] OR struthioniformes[tiab] OR ferina[tiab] OR sanderling[tiab] OR sanderlings[tiab] OR spheniscidae[tiab] OR cuttlefishes[tiab] OR cygnet[tiab] OR dasycneme[tiab] OR gadwall[tiab] OR gadwalls[tiab] OR pelobates fuscus[tiab] OR wryneck[tiab] OR wrynecks[tiab] OR afrosoricida[tiab] OR culaea[tiab] OR dover sole[tiab] OR dover soles[tiab] OR paralichthyidae[tiab] OR passeridae[tiab] OR osteolaemus[tiab] OR song thrushes[tiab] OR bluethroat[tiab] OR bluethroats[tiab] OR hydrophiidae[tiab] OR megrim[tiab] OR mephitidae[tiab] OR strepsirhini[tiab] OR tomistoma[tiab] OR epidalea[tiab] OR osmeriformes[tiab] OR bush babies[tiab] OR tarsiiform[tiab] OR atelinae[tiab] OR bufotes[tiab] OR eurasian coot[tiab] OR eurasian coots[tiab] OR galagidae[tiab] OR geopelia[tiab] OR philomachus[tiab] OR tubulidentata[tiab] OR bombinatoridae[tiab] OR pelobatidae[tiab] OR tachysurus[tiab] OR ailuridae[tiab] OR woodlark[tiab] OR woodlarks[tiab] OR alcelaphinae[tiab] OR redshank[tiab] OR redshanks[tiab] OR salientia[tiab] OR sand smelt[tiab] OR sand smelts[tiab] OR woodmice[tiab] OR woodmouse[tiab] OR dasyproctidae[tiab] OR eurasian wigeon[tiab] OR eurasian wigeons[tiab] OR garganey[tiab] OR garganeys[tiab] OR lemon sole[tiab] OR lemon soles[tiab] OR common dab[tiab] OR common dabs[tiab] OR graylag[tiab] OR graylags[tiab] OR leucorodia[tiab] OR osphronemidae[tiab] OR bewickii[tiab] OR common moorhen[tiab] OR common moorhens[tiab] OR decapodiformes[tiab] OR gobbler[tiab] OR gobblers[tiab] OR odontophoridae[tiab] OR paddlefishes[tiab] OR eutheria[tiab] OR salmonine[tiab] OR esociformes[tiab] OR eurasian woodcock[tiab] OR eurasian woodcocks[tiab] OR european smelt[tiab] OR european smelts[tiab] OR goldfishes[tiab] OR tenches[tiab] OR tyranni[tiab] OR common chaffinch[tiab] OR common chaffinchs[tiab] OR common redstart[tiab] OR common redstarts[tiab] OR common roach[tiab] OR common roachs[tiab] OR great knot[tiab] OR great knots[tiab] OR potoroidae[tiab] OR alytidae[tiab] OR coregonine[tiab] OR dipteral[tiab] OR leveret[tiab] OR poeciliopsis gracilis[tiab] OR amphiumidae[tiab] OR batrachoidiformes[tiab] OR bighead goby[tiab] OR heteropneustidae[tiab] OR lullula[tiab] OR norway pout[tiab] OR norway pouts[tiab] OR sipunculida[tiab] OR dogfishes[tiab] OR sebastidae[tiab] OR tarsiidae[tiab] OR alethinophidia[tiab] OR common nase[tiab] OR common nases[tiab] OR common sandpiper[tiab] OR common sandpipers[tiab] OR eurasian blackcap[tiab] OR eurasian blackcaps[tiab] OR pterocnemia[tiab] OR syngnathiformes[tiab] OR common chaffinches[tiab] OR eupleridae[tiab] OR octopodiformes[tiab] OR phascolarctidae[tiab] OR scophthalmidae[tiab] OR starry smooth-hound[tiab] OR starry smooth-hounds[tiab] OR whitefishes[tiab] OR cuniculidae[tiab] OR european sprat[tiab] OR european sprats[tiab] OR rosy bitterling[tiab] OR rosy bitterlings[tiab] OR common dace[tiab] OR common daces[tiab] OR lesser weever[tiab] OR lesser weevers[tiab] OR scaldfish[tiab] OR water rail[tiab] OR water rails[tiab] OR alouattinae[tiab] OR centrarchiformes[tiab] OR common whitethroat[tiab] OR common whitethroats[tiab] OR gavialidae[tiab] OR grey gurnard[tiab] OR grey gurnards[tiab] OR lateolabracidae[tiab] OR rheiformes[tiab] OR tub gurnard[tiab] OR tub gurnards[tiab] OR common chiffchaff[tiab] OR common chiffchaffs[tiab] OR garfishes[tiab] OR lesser whitethroat[tiab] OR lesser whitethroats[tiab] OR myoxidae[tiab] OR seabasses[tiab] OR spariformes[tiab] OR umbridae[tiab] OR yellow boxfish[tiab] OR anabantiformes[tiab] OR aotidae[tiab] OR common bleak[tiab] OR common bleaks[tiab] OR common rudd[tiab] OR common rudds[tiab] OR greater pipefish[tiab] OR hapale[tiab] OR nandiniidae[tiab] OR stone loaches[tiab] OR whinchat[tiab] OR whinchats[tiab] OR acanthuriformes[tiab] OR brotula barbata[tiab] OR common ling[tiab] OR common lings[tiab] OR common roaches[tiab] OR cottonrat[tiab] OR cottonrats[tiab] OR douroucoulis[tiab] OR dromaiidae[tiab] OR fitches[tiab] OR fitchew[tiab] OR galaxiiformes[tiab] OR laprine[tiab] OR saimiriinae[tiab] OR solenette[tiab] OR tarsii[tiab] OR tompot blenny[tiab] OR common dragonet[tiab] OR common dragonets[tiab] OR longspined bullhead[tiab] OR longspined bullheads[tiab] OR monotremate[tiab] OR monotremates[tiab] OR pempheriformes[tiab] OR perdicinae[tiab] OR presbytini[tiab] OR smegmamorpha[tiab] OR bighead gobies[tiab] OR carangaria incertae sedis[tiab] OR coiidae[tiab] OR fivebeard rockling[tiab] OR foulmart[tiab] OR foumart[tiab] OR grasskeet[tiab] OR greater pipefishes[tiab] OR ibices[tiab] OR millionfish[tiab] OR muguliformes[tiab] OR norwegian topknot[tiab] OR peewit[tiab] OR red sea sailfin tang[tiab] OR rupicapras[tiab] OR sheatfishes[tiab] OR tompot blennies[tiab] OR twait shad[tiab] OR yellow boxfishes[tiab]) NOT medline[sb])

*EMBASE search AVM component AVM component*

Exp cerebrovascular malformation/ OR Rendu Osler Weber disease/ OR ((brain arteriovenous malformation* OR AVM OR bAVM OR Arteriovenous Aneurysm* OR (Arteriovenous Malformation AND Brain) OR aneurysm of the vein of Galen OR aneurysms of the vein of Galen OR Galen vein aneurysm OR great brain vein aneurysm OR malformation of the vein of Galen OR malformations of the vein of Galen OR vein of Galen aneurysm OR vein of Galen aneurysmal malformation OR vein of Galen aneurysmal malformations OR vein of Galen aneurysms OR

vein of Galen arteriovenous malformation OR vein of Galen arteriovenous malformations OR vein of Galen malformation OR vein of Galen malformations OR VGAM OR VGM OR (angiomatosis AND hereditary haemorrhagic) OR (angiomatosis AND hereditary hemorrhagic) OR

(disease AND osler rendu weber) OR (disease AND rendu osler weber) OR

(haemorrhagic angiomatosis AND hereditary) OR (haemorrhagic telangiectasia AND hereditary) OR (hemorrhagic angiomatosis AND hereditary) OR (hemorrhagic telangiectasia AND hereditary) OR hereditary haemorrhagic angiomatosis OR hereditary haemorrhagic telangiectasia OR hereditary haemorrhagic telangiectasis OR hereditary hemorrhagic angiomatosis OR hereditary hemorrhagic telangiectasia OR hereditary hemorrhagic telangiectasis OR osler rendu weber disease OR osler rendu weber syndrome OR osler weber rendu disease OR rendu osler angiomatosis OR rendu osler disease OR Rendu Osler Weber syndrome OR rendu weber osler disease OR Rendu-Osler-Webers disease OR (syndrome AND osler rendu weber) OR (telangiectasia AND hereditary haemorrhagic) OR (telangiectasia AND hereditary hemorrhagic) OR (telangiectasis AND hereditary haemorrhagic) OR (telangiectasis AND hereditary hemorrhagic) OR teleangiectasia hereditaria haemorrhagica OR weber rendu osler disease).ti,ab,kw.)

*EMBASE SYRCLE Animal Filter*

exp animal experiment/ or exp animal model/ or exp experimental animal/ or exp transgenic animal/ or exp male animal/ or exp female animal/ or exp juvenile animal/ OR animal/ OR chordata/ OR vertebrate/ OR tetrapod/ OR exp fish/ OR amniote/ OR exp amphibia/ OR mammal/ OR exp reptile/ OR exp sauropsid/ OR therian/OR exp monotremate/ OR placental mammals/ OR exp marsupial/ OR Euarchontoglires/ OR exp Afrotheria/ OR exp Boreoeutheria/ OR exp Laurasiatheria/ OR exp Xenarthra/ OR primate/ OR exp Dermoptera/ OR exp Glires/ OR exp Scandentia/ OR Haplorhini/ OR exp prosimian/ OR simian/ OR exp tarsiiform/ OR Catarrhini/ OR exp Platyrrhini/ OR ape/ OR exp Cercopithecidae/ OR hominid/ OR exp hylobatidae/ OR exp chimpanzee/ OR exp gorilla/ OR exp orang utan/ OR (animal OR animals OR pisces OR fish OR fishes OR catfish OR catfishes OR sheatfish OR silurus OR arius OR heteropneustes OR clarias OR gariepinus OR fathead minnow OR fathead minnows OR pimephales OR promelas OR cichlidae OR trout OR trouts OR char OR chars OR salvelinus OR salmo OR oncorhynchus OR guppy OR guppies OR millionfish OR poecilia OR goldfish OR goldfishes OR carassius OR auratus OR mullet OR mullets OR mugil OR curema OR shark OR sharks OR cod OR cods OR gadus OR morhua OR carp OR carps OR cyprinus OR carpio OR killifish OR eel OR eels OR anguilla OR zander OR sander OR lucioperca OR stizostedion OR turbot OR turbots OR psetta OR flatfish OR flatfishes OR plaice OR pleuronectes OR platessa OR tilapia OR tilapias OR oreochromis OR sarotherodon OR common sole OR dover sole OR solea OR zebrafish OR zebrafishes OR danio OR rerio OR seabass OR dicentrarchus OR labrax OR morone OR lamprey OR lampreys OR petromyzon OR pumpkinseed OR pumpkinseeds OR lepomis OR gibbosus OR herring OR clupea OR harengus OR amphibia OR amphibian OR amphibians OR anura OR salientia OR frog OR frogs OR rana OR toad OR toads OR bufo OR xenopus OR laevis OR bombina OR epidalea OR calamita OR salamander OR salamanders OR newt OR newts OR triturus OR reptilia OR reptile OR reptiles OR bearded dragon OR pogona OR vitticeps OR iguana OR iguanas OR lizard OR lizards OR anguis fragilis OR turtle OR turtles OR snakes OR snake OR aves OR bird OR birds OR quail OR quails OR coturnix OR bobwhite OR colinus OR virginianus OR poultry OR poultries OR fowl OR fowls OR chicken OR chickens OR gallus OR zebra finch OR taeniopygia OR guttata OR canary OR canaries OR serinus OR canaria OR parakeet OR parakeets OR grasskeet OR parrot OR parrots OR psittacine OR psittacines OR shelduck OR tadorna OR goose OR geese OR branta OR leucopsis OR woodlark OR lullula OR flycatcher OR ficedula OR hypoleuca OR dove OR doves OR geopelia OR cuneata OR duck OR ducks OR greylag OR graylag OR anser OR harrier OR circus pygargus OR red knot OR great knot OR calidris OR canutus OR godwit OR limosa OR lapponica OR meleagris OR gallopavo OR jackdaw OR corvus OR monedula OR ruff OR philomachus OR pugnax OR lapwing OR peewit OR plover OR vanellus OR swan OR cygnus OR columbianus OR bewickii OR gull OR chroicocephalus OR ridibundus OR albifrons OR great tit OR parus OR aythya OR fuligula OR streptopelia OR risoria OR spoonbill OR platalea OR leucorodia OR blackbird OR turdus OR merula OR blue tit OR cyanistes OR pigeon OR pigeons OR columba OR pintail OR anas OR starling OR sturnus OR owl OR athene noctua OR pochard OR ferina OR cockatiel OR nymphicus OR hollandicus OR skylark OR alauda OR tern OR sterna OR teal OR crecca OR oystercatcher OR haematopus OR ostralegus OR shrew OR shrews OR sorex OR araneus OR crocidura OR russula OR european mole OR talpa OR chiroptera OR bat OR bats OR eptesicus OR serotinus OR myotis OR dasycneme OR daubentonii OR pipistrelle OR pipistrellus OR cat OR cats OR felis OR catus OR feline OR dog OR dogs OR canis OR canine OR canines OR otter OR otters OR lutra OR badger OR badgers OR meles OR fitchew OR fitch OR foumart or foulmart OR ferrets OR ferret OR polecat OR polecats OR mustela OR putorius OR weasel OR weasels OR fox OR foxes OR vulpes OR common seal OR phoca OR vitulina OR grey seal OR halichoerus OR horse OR horses OR equus OR equine OR equidae OR donkey OR donkeys OR mule OR mules OR pig OR pigs OR swine OR swines OR hog OR hogs OR boar OR boars OR porcine OR piglet OR piglets OR sus OR scrofa OR llama OR llamas OR lama OR glama OR deer OR deers OR cervus OR elaphus OR cow OR cows OR bos taurus OR bos indicus OR bovine OR bull OR bulls OR cattle OR bison OR bisons OR sheep OR sheeps OR ovis aries OR ovine OR lamb OR lambs OR mouflon OR mouflons OR goat OR goats OR capra OR caprine OR chamois OR rupicapra OR leporidae OR lagomorpha OR lagomorph OR rabbit OR rabbits OR oryctolagus OR cuniculus OR laprine OR hares OR lepus OR rodentia OR rodent OR rodents OR murinae OR mouse OR mice OR mus OR musculus OR murine OR woodmouse OR apodemus OR rat OR rats OR rattus OR norvegicus OR guinea pig OR guinea pigs OR cavia OR porcellus OR hamster OR hamsters OR mesocricetus OR cricetulus OR cricetus OR gerbil OR gerbils OR jird OR jirds OR meriones OR unguiculatus OR jerboa OR jerboas OR jaculus OR chinchilla OR chinchillas OR beaver OR beavers OR castor fiber OR castor canadensis OR sciuridae OR squirrel OR squirrels OR sciurus OR chipmunk OR chipmunks OR marmot OR marmots OR marmota OR suslik OR susliks OR spermophilus OR cynomys OR cottonrat OR cottonrats OR sigmodon OR vole OR voles OR microtus OR myodes OR glareolus OR primate OR primates OR prosimian OR prosimians OR lemur OR lemurs OR lemuridae OR loris OR bush baby OR bush babies OR bushbaby OR bushbabies OR galago OR galagos OR anthropoidea OR anthropoids OR simian OR simians OR monkey OR monkeys OR marmoset OR marmosets OR callithrix OR cebuella OR tamarin OR tamarins OR saguinus OR leontopithecus OR squirrel monkey OR squirrel monkeys OR saimiri OR night monkey OR night monkeys OR owl monkey OR owl monkeys OR douroucoulis OR aotus OR spider monkey OR spider monkeys OR ateles OR baboon OR baboons OR papio OR rhesus monkey OR macaque OR macaca OR mulatta OR cynomolgus OR fascicularis OR green monkey OR green monkeys OR chlorocebus OR vervet OR vervets OR pygerythrus OR hominoidea OR ape OR apes OR hylobatidae OR gibbon OR gibbons OR siamang OR siamangs OR nomascus OR symphalangus OR hominidae OR orangutan OR orangutans OR pongo OR chimpanzee OR chimpanzees OR pan troglodytes OR bonobo OR bonobos OR pan paniscus OR gorilla OR gorillas OR troglodytes).ti,ab.

Supplementary Figure 1. Rupture forest plot.

Supplementary Figure 2. Survival forest plot.
